# Genetic Polymorphisms and Phenotypic Profiles of Sulfadiazine-Resistant and Sensitive *Toxoplasma gondii* Isolates Obtained from Newborns with Congenital Toxoplasmosis in Minas Gerais, Brazil

**DOI:** 10.1371/journal.pone.0170689

**Published:** 2017-01-24

**Authors:** Letícia Azevedo Silva, João Luís Reis-Cunha, Daniella Castanheira Bartholomeu, Ricardo Wagner Almeida Vítor

**Affiliations:** 1 Laboratório de Toxoplasmose, Departamento de Parasitologia, Instituto de Ciências Biológicas, Universidade Federal de Minas Gerais, Belo Horizonte, Minas Gerais, Brasil; 2 Laboratório de Genômica de Parasitos, Departamento de Parasitologia, Instituto de Ciências Biológicas, Universidade Federal de Minas Gerais, Belo Horizonte, Minas Gerais, Brasil; Yeshiva University Albert Einstein College of Medicine, UNITED STATES

## Abstract

**Background:**

Previous *Toxoplasma gondii* studies revealed that mutations in the *dhps* (*dihydropteroate synthase*) gene are associated with resistance to sulfonamides. Although Brazilian strains are genotypically different, very limited data are available regarding the susceptibility of strains obtained from human to sulfonamides. The aim of this study was to evaluate the efficacy of sulfadiazine (SDZ) against Brazilian isolates of *T*. *gondii* and verify whether isolates present polymorphisms in the *dhps* gene. We also investigated whether the virulence-phenotype and/or genotype were associated with the profile of susceptibility to SDZ.

**Methods:**

Five *T*. *gondii* isolates obtained from newborns with congenital toxoplasmosis were used to verify susceptibility. Mice were infected with 10^4^ tachyzoites and orally treated with different doses of SDZ. The mortality curve was evaluated by the Log-rank test. The presence of polymorphisms in the *dhps* gene was verified using sequencing. A descriptive analysis for 11 Brazilian isolates was used to assess the association between susceptibility, genotype, and virulence-phenotype.

**Results:**

Statistical analysis showed that TgCTBr03, 07, 08, and 16 isolates were susceptible to SDZ, whereas TgCTBr11 isolate presented a profile of resistance to SDZ. Nineteen polymorphisms were identified in *dhps* exons. Seven polymorphisms corresponded to non-synonymous mutations, with four being new mutations, described for the first time in this study. No association was found between the profile of susceptibility and the virulence-phenotype or genotype of the parasite.

**Conclusions:**

There is a high variability in the susceptibilities of Brazilian *T*. *gondii* strains to SDZ, with evidence of drug resistance. Despite the large number of polymorphisms identified, the profile of susceptibility to SDZ was not associated with any of the *dhps* variants identified in this study. Other genetic factors, not yet determined, may be associated with the resistance to SDZ; thus, further studies are needed as a basis for a more adequate toxoplasmosis treatment.

## Introduction

*Toxoplasma gondii* is an obligate intracellular protozoan parasite distributed worldwide that infects a wide range of warm-blooded animals [1]. Infection in humans is usually asymptomatic, but a severe manifestation can occur in cases of congenital toxoplasmosis and immune-compromised individuals, with treatment being indicated in such cases [1].

Treatment of toxoplasmosis usually uses a combination of sulfadiazine (SDZ) and pyrimethamine, which demonstrate a remarkable synergistic activity against the replication of tachyzoites through the sequential inhibition of parasite dihydropteroate synthase (DHPS) and dihydrofolate reductase (DHFR). These two enzymes are responsible for the synthesis of the folate compounds essential for *T*. *gondii* survival and replication [2]. However, failures in the toxoplasmosis treatment have been reported in the literature, especially in immunocompromised individuals and in cases of congenital transmission [2, 3]. These failures may be related to host factors or to parasite factors [4]. Host factors, for example, are malabsorption or drug intolerance. Parasite factors may be differences in drug-susceptibility between genetically different *T*. *gondii* strains or the development of drug resistance caused by mutations in the target gene [3, 4].

Previous studies have shown that mutations in the genes encoding antifolate targets normally lead to resistance to these drugs. A study using clinical samples obtained in the UK showed the presence of six mutations within the *T*. *gondii dhps* gene. Mutation N407D, which is equivalent to the 437 position in *Plasmodium*, was reported as being associated with sulfonamide resistance in one clinical isolate [5]. This mutation was also retrieved in a laboratory-induced sulfamethoxazole-resistant strain [6].

Recently, the susceptibility of 17 *T*. *gondii* isolates obtained in France was evaluated with the following three anti-toxoplasmic drugs: sulfadiazine, pyrimethamine, and atovaquone. Some variability was verified for the susceptibility of *T*. *gondii* strains to pyrimethamine and atovaquone, but with no clear evidence of drug resistance. On the other hand, a high variability was found in the susceptibility to sulfadiazine, and three strains resistant to this drug were identified. In addition, a new mutation was identified in the *dhps* gene of *T*. *gondii* (A587V), which was associated to resistance of one of the strains to sulfadiazine [7].

Studies on susceptibility to chemotherapy in experimental toxoplasmosis have been predominantly conducted using strains belonging to three genetic clonal lineages: Types I, II, or III, common in Europe and North America. However, studies using multi-locus markers showed a higher genetic diversity of *T*. *gondii* in South America than in the Northern Hemisphere. In Brazil, there is a predominance of atypical genotypes, with the following four genotypes being considered common lineages: BrI BrII, BrIII, and BrIV [8, 9]. Little is known about the effects of drugs on atypical strains, such as those found in South America. The fact that Brazilian *T*. *gondii* strains have a different population structure and a greater genetic diversity as well as being more virulent than the clonal lineages, can lead to different susceptibility profiles to chemotherapy of Brazilian isolates compared to isolates from the Northern Hemisphere. Thus, further studies using Brazilian isolates need to be conducted.

The objectives of this study were to evaluate the efficacy of sulfadiazine on Brazilian clinical isolates of *T*. *gondii*; to verify whether these isolates present polymorphisms in the antifolates resistance-associated gene *dhps*; and to assess whether other factors, such as parasite genotype and virulence-phenotype, could be associated with the profile of susceptibility to sulfadiazine. Despite the large number of polymorphisms identified, there was no clear evidence of association with the profile of resistance to sulfadiazine.

## Materials and Methods

### *Toxoplasma gondii* isolates

Five *T*. *gondii* isolates were used to assess susceptibility to sulfadiazine: TgCTBr03, TgCTBr07, TgCTBr08, TgCTBr11, and TgCTBr16. They were previously obtained by mouse bioassay of blood obtained from newborns with congenital toxoplasmosis in Minas Gerais state, Brazil [10], under parental informed consent. The protocols used in this previous study [10] were approved by the local Human Research Ethics Committee (COEP-Federal University of Minas Gerais, protocol 298/06). These isolates are maintained cryopreserved in Dimethylsulfoxide (DMSO) in our laboratory, as previously described [11]. Clinical data of these newborns are summarized in Table 1 (age at the time of blood collection, gender, major clinical signs, confirmative serologic results and *T*. *gondii* genotype based on Polymerase chain reaction- Restriction fragment length polymorphism (PCR-RFLP)).The TgCTBr16 isolate was previously genotyped by Pinheiro *et al*. [12] and the other isolates were genotyped by Carneiro *et al*. [10].

**Table 1:** *Toxoplasma gondii* isolates tested for susceptibility to Sulfadiazine and characteristics of the newborns from which the isolates were obtained.

Isolate	Patient gender	Age^a^	Clinical signs at birth	Clinical signs 12 mo. after treatment	IgM screening^e^	ELFA-IgG^f^	PCR-RFLP Genotype
TgCTBr03	Male	52	No^b^	No	+	+	#206
TgCTBr07	Female	43	ACRL	CRL	+	+	#67
TgCTBr08	Male	41	ACRL	CRL	+	+	#11 (Br II)
TgCTBr11	Female	78	ARL^c^	ND^d^	+	+	#11 (Br II)
TgCTBr16	Male	60	CRL	CRL	+	+	#8 (Br III)

^a^ Age (days) of newborn when *T*. *gondii* was isolated

^b^ No clinical signs

^c^ This child also had hepatomegaly, splenomegaly, microphthalmia

^d^ND- not done because this child died at 4 months of age

^e^ Anti-*T*. *gondii* IgM antibodies in blood on filter paper after birth, using the TOXO IgM kit (Q-Preven®, Symbiosis, Leme, Brazil)

^f^ Anti-*T*. *gondii* IgG by the enzyme-linked fluorometric assay (ELFA-VIDAS®, bioMérrieux SA, Lyon, France)

ARL, active retinochoroidal lesions; CRL, cicatricial retinochoroidal lesions; ACRL, active and cicatricial retinochoroidal lesions.

### Assay of susceptibility to sulfadiazine (SDZ)

Fresh tachyzoites were obtained from the peritoneal cavities of Swiss mice after thawing, DMSO removal with phosphate buffered saline (PBS) pH 7.2, and intraperitoneal inoculums, as previously described [11]. The number of tachyzoites present in the peritoneal aspirate was counted in a Neubauer chamber under optical microscope and diluted in PBS pH 7.2 to obtain the desired number of parasites.

Mice were obtained from Center of Bioterism (CEBIO) of the Institute of Biological Sciences–Universidade Federal de Minas Gerais (UFMG). Female Swiss Webster mice, six to eight weeks of age were used in the experimental groups. Mice were housed 10 per cage, according to temperature, humidity, and lighting standards of the Conselho Nacional de Controle de Experimentação Animal (CONCEA)–Brazil, with ad libitum food (Nuvilab®, CR1, Nuvital, Brazil), and ad libitum water. Cages were composed of polypropylene, approximately 41x34x16cm in size, and changed weekly.

The number of ten Swiss mice was used per experimental group, according to Khan *et al*. (1997) [13]. Mice were intraperitoneally infected (i.p.) with 10^4^ tachyzoites from each *T*. *gondii* isolate. SDZ (Catarinense, Brazil) was dissolved in Carboxymethylcellulose (0.25%) to achieve the desired concentration. Treatment with 80, 160, or 320 mg/Kg/day of SDZ administered by gavage was initiated 48 hours post-infection and continued for 10 days. A group of 10 infected mice was maintained as a non-treated control (NTC).

The mice were followed for 30 days post-infection (DPI), twice a day, to assess the efficacy of the therapeutic treatments. Some infected animals, after going through an initial period of weakness (that may include rapid weight loss, ruffled fur and animal prostration), suddenly recover, gain weight and do not succumb to acute infection, therefore we used death of mice as an endpoint. Death is a required endpoint for our survival experiments to determine the actual number of animals that died spontaneously due to therapeutic failure of SDZ. No analgesics and anesthetics were used during follow-up since drug interactions with other medications can alter the results of experiments. Only SDZ (the drug of choice for the treatment of human toxoplasmosis) was administered as previously described. All deaths were due to toxoplasmosis.

The mice that had survived until the end of the experiment were euthanized by cervical dislocation according to CONCEA guidelines. The survival rates, the presence of brain cysts (cyst count in the optical microscope) and specific IgG antibody by enzyme-linked immunosorbent assay (ELISA) in the surviving mice were analyzed as previously described [14]. If no cysts were observed, the brain homogenate was i.p. inoculated into an uninfected mouse (bioassay). The sub-inoculated mice were followed for 30 DPI.

The mortality of mice was analyzed using the Log-rank (Mantel-Cox) test to compare the survival curves and the efficacy of the treatments [15]. The statistical differences among the groups were verified using the non-parametric Mann-Whitney or Kruskal-Wallis tests. The mean number of brain cysts in the surviving mice was analyzed using the non-parametric Kruskal-Wallis test (p < 0.05).

### Association between susceptibility to SDZ and genotype or virulence of *T*. *gondii*

A descriptive analysis was also performed to verify the association between the profile of susceptibility to SDZ defined in this study and the molecular (genotype) or biological (virulence for mice) characteristics of the isolates. The results from the five *T*. *gondii* isolates were compared with previous SDZ susceptibility results from six Brazilian *T*. *gondii* isolates: two obtained from humans (SAF and EGS), two from dogs (D4 and D7), and two from free-range chickens (CH1 and CH3) [14]. A composite dataset of the 11 isolates was constructed. The survival rates were used as criteria for comparison because the survival curves of these six isolates were not analyzed by the Log-rank (Mantel-Cox) test. The *T*. *gondii* isolates were classified as highly susceptible (mice survival rates higher than or equal to 80%, regardless of the SDZ dosage used); resistant (mice survival rates between 0% and 40% after treatment); and intermediary (mice survival rates between 40% and 80% after treatment).

Genotyping, virulence in mice, and the allele type at the CS3 marker data were retrieved from previously published reports from our laboratory [9, 10].

### Sequencing of the *dhps* gene

*T*. *gondii* tachyzoites from each isolate were obtained from the peritoneal cavities of Swiss mice as previously described [16]. Fresh tachyzoites were submitted for DNA extraction using the kit Wizard® Genomic DNA Purification (Promega), according to the manufacturer’s instructions.

The target DNA sequence of the *dhps* gene was amplified by PCR (Polymerase chain reaction). The previously described internal primers were used to amplify the six *dihydropteroate synthase* (*dhps*) exons [5]. No previous amplification by the external primers was necessary because the DNA was extracted from purified tachyzoites. The previously described primers to amplify the *dhps* exon 1 [5] failed to completely amplify this exon, leaving the initial part of the protein uncovered (from amino acid 1 to 388). New primers were designed to amplify this region using the software Oligo Explorer v. 1.5 by Gene Link. The *dhps* exon 1 was amplified as two overlapping fragments because of its large size: exon 1a (primers: *dhps* exon 1a New F, 5´-ACGGATATGAGGAGCGCTAC-3´; *dhps* exon 1a New R, 5´-GAAGCAGCTCCTTCACAGAC-3´) and exon 1b (primers: *dhps* exon 1b New F, 5´-GTTATACACCCTGATGTGCG-3´; *dhps* exon 1b New R, 5´-GCATGGCAAAATAGACCGTC-3´).

The amplification reactions were performed at a final volume of 100 μL, containing 10 μL 10X High Fidelity PCR Buffer (Invitrogen), 250 mM MgSO_4_, 25 mM of each deoxynucleotide (dATP/dTTP/dGTP/dCTP; Invitrogen), 3.5 U of Platinum® Taq DNA Polymerase High Fidelity (Invitrogen), 50 pmol of each primer, and 5 μL of DNA. A negative control (without DNA) was included in each reaction mixture. Genomic DNA from RH (type I), ME49 (type II), and VEG (type III) strains was used as a control. The first amplification step consisted of 3 min of denaturation at 94°C, 50 cycles with denaturation at 94°C for 20 s, annealing at primer-dependent temperatures for 20 s, and extension at 68°C for 90 s. A final extension step was performed at 68°C for 5 min. PCR products were resolved by 1% agarose gel electrophoresis. The PCR products were recovered from the gel and purified using a PCR purification kit (NucleoSpin® Extract II–Nucleic Acid and Protein Purification—Macherey-Nagel). DNA amplified from each sample (1000 ng) was lyophilized and sequenced at both ends in the *ABI Prism 3730xl DNA Analyser* (Applied Biosystems) by Macrogen Inc (Korea).

### Analysis of the sequences of the *dhps* gene

The forward and reverse sequences obtained from each exon were processed using Phred and CAP3 [17]. Bases with a phred score lower than 20 were removed (PMID:9521922). Strain polymorphisms were analyzed by alignment of the contig sequences using the multiple sequence alignment program ClustalW (http://www.ebi.ac.uk/Tools/msa/clustalw2/). The sequences of the strains GT1 (type I), ME49 (type II), and VEG (type III) obtained from the ToxoDB database (http://www.toxodb.org) were used as reference. The protein sequences of each isolate were compared using tblastn (Translated BLAST, http://www.blast.ncbi.nlm.gov/) translated by ExPASy Tools Translate (http://www.expasy.org/tools/) and submitted to multiple alignments using ClustalW.

### Ethical statement

This study was carried out in strict accordance with the recommendations of the Conselho Nacional de Controle de Experimentação Animal (CONCEA)—Brazil. The protocol conducted in this study was approved by the Ethics Committee in Animal Experimentation (CETEA) of the Universidade Federal de Minas Gerais, Brazil (Protocol CEUA 257/2012). All efforts were made to minimize suffering. The euthanasia method conducted in this study has been properly approved by CETEA/UFMG.

## Results

### Susceptibility to Sulfadiazine (SDZ)

**Fig 1:**
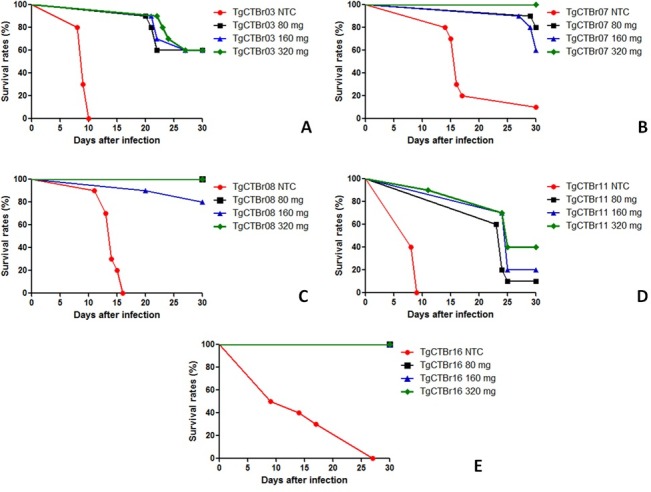
Survival rates of *Swiss* mice infected with 10^4^ tachyzoites from *T*. *gondii* isolates and treated during 10 days with different doses of sulfadiazine (80, 160, and 320 mg/Kg/day), initiating 48 hours after the inoculum. Isolates: TgCTBr03 (A), TgCTBr07 (B), TgCTBr08 (C), TgCTBr11 (D) and TgCTBr16. NTC Groups (E): non-treated control groups, infected with the respective isolate.

The survival rates observed in the mice infected with the TgCTBr03 isolate were 0% for the non-treated control (NTC) group and 60% for the groups treated with the different SDZ dosages (Fig 1A). For the TgCTBr07 isolate, the survival rates were 10, 80, 60 and 100% for the NTC and 80, 160, and 320 mg/Kg/day SDZ groups respectively. (Fig 1B). For the TgCTBr08 isolate, the survival rates were 0, 100, 80, and 100% for the NTC and 80, 160, and 320 mg/Kg/day SDZ groups respectively (Fig 1C). For the TgCTBr11 isolate, the survival rates were 0, 10, 20, and 40%, respectively (Fig 1D), and for TgCTBr16 isolate, the survival rates were 0, 100, 100, and 100%, respectively (Fig 1E).

A significant difference (Log-rank test) was observed when the survival curves of the groups treated with different SDZ dosages were compared to the survival curve of the NTC group (TgCTBr03 (p = 0.0139), TgCTBr07 (p = 0.0003), TgCTBr08 (p<0.0001), and TgCTBr16 (p<0.0001)). In these cases, the treatment significantly increased the survival rate of the infected mice. For the TgCTBr11 isolate, the Log-rank test showed that there is no statistical difference between the survival curves of the SDZ treated groups and the survival curve of the NTC group (p = 0.1161). All surviving mice at 30 DPI presented anti-*T*. *gondii* IgG antibodies. No significant differences were observed in the antibody levels or the number of brain cysts of these animals when the groups treated with different dosages of SDZ were compared (p>0.05).

It was necessary to conduct a bioassay of one surviving mouse inoculated with TgCTBr08 isolate, six inoculated with TgCTBr11 isolate, and 30 inoculated with TgCTBr16 isolate because these animals did not present cerebral cysts by optical microscopy at 30 DPI (Table 2), despite testing positive by ELISA. The animal sub-inoculated with TgCTBr08 isolate died of an acute infection after an 11-day follow up, confirming parasitism. After the bioassay with the TgCTBr11 isolate, one animal from the 160 mg/Kg/day group died due to acute infection 10 days after being sub-inoculated, and the other animal survived the 30-day follow-up without presenting cerebral cysts or anti-*T*. *gondii* IgG antibodies (Table 2). The four mice in the 320 mg/Kg/day group survived the bioassay and did not present cerebral cysts or antibodies. The mortality rates (%) for the bioassay using the TgCTBr16 isolate were 60, 40, and 50 for the 80, 160, and 320 mg/Kg/day groups, respectively. These animals died of an acute infection before day 17 follow-up. The surviving animals did not present cerebral cysts or antibodies, except for one in the 80 mg/Kg/day group, which was ELISA positive for *T*. *gondii* (Table 2).

**Table 2:** Bioassay of the surviving mice after infection with *T*. *gondii* isolates TgCTBr08, TgCTBr11, and TgCTBr16 and treatment with different doses of SDZ, for verification of cerebral parasitism.

Isolate	Origin group	Mortality^a^	Presence of cysts^b^	Seropositivity (ELISA)^c^
TgCTBr08	80 mg/Kg/day	1/1 (100%)	ND	ND
TgCTBr11	160 mg/Kg/day	1/2 (50%)	0/1	0/1
	320 mg/Kg/day	0/4 (0%)	0/4	0/4
TgCTBr16	80 mg/Kg/day	6/10 (60%)	0/4	1/4
	160 mg/Kg/day	4/10 (40%)	0/6	0/6
	320 mg/Kg/day	5/10 (50%)	0/5	0/5

^a^ number of dead mice/total of bio-assayed mice.

^b^ number of mice that presented cerebral cysts/total of bioassay survivors

^c^ number of mice with antibodies IgG ant-*T*. *gondii*/ total of bioassay survivors

ND–Not done.

### Association between susceptibility to SDZ and *T*. *gondii* genotype or virulence

A descriptive analysis of the results for the 11 Brazilian *T*. *gondii* isolates showed that there was no association between parasite genotype and susceptibility to SDZ because the three isolates belonging to genotype ToxoDB #11 (TgCTBr08, TgCTBr11, and D4) presented different susceptibility profiles (Table 3). This analysis could not be performed with the other isolates because each one belonged to a different genotype. For the isolates with intermediary susceptibility and for the highly susceptible isolates, no association was observed between the profile of susceptibility to SDZ and the virulence-phenotype in mice, nor with the allele type at the CS3 locus because the isolates displaying the same profile have different degrees of virulence. The two isolates with a profile of resistance to SDZ (TgCTBr11 and EGS) presented a virulent-phenotype for mice as well as the allele type I at the CS3 locus (Table 3).

**Table 3:** Genotypic and phenotypic profile of 11 Brazilian *Toxoplasma gondii* isolates with different susceptibilities to sulfadiazine.

Isolate	Untreated survivors (%)	Survivors with 80 mg/Kg (%)^a^	Survivors with 160 mg/Kg (%)	Survivors with 320 mg/Kg (%)	Toxo DB Genotype^b^	Mice virulence	Allele type at the CS3 locus^c^	Profile of susceptibility to SDZ
TgCTBr03	0	60	60	60	#206	Virulent	II	Intermediate susceptibility
TgCTBr07	10	80	60	100	#67	Intermediate	II	Intermediate susceptibility
TgCTBr08	0	100	80	100	#11 (BrII)	Virulent	I	Highly susceptible
TgCTBr11	0	10	20	40	#11 (BrII)	Virulent	I	Resistant
TgCTBr16	0	100	100	100	#08 (BrIII)	Intermediate	III	Highly susceptible
SAF	0	60	80	60	#108	Virulent	I	Intermediate susceptibility^*^
EGS	0	0	10	20	#229	Virulent	I	Resistant^*^
D4	10	100	90	100	#11 (BrII)	Intermediate	I	Highly susceptible^*^
D7	10	80	90	90	#228	Intermediate	III	Highly susceptible^*^
CH1	20	80	100	100	#19	Intermediate	III	Intermediate susceptibility^*^
CH3	0	40	60	80	#163	Intermediate	II	Highly susceptible^*^

^a^percentage of surviving mice after infection with *Toxoplama gondii* isolates and treatment with sulfadiazine

^b^Genotyped by Carneiro *et al*.[10] and Silva *et al*. [9]

^c^allele type according to Silva *et al*. [9].

*According previous study by Alves & Vitor [14].

### Sequencing of the *dhps* gene

Nucleotide sequence data reported in this paper are available in the GenBank database under the accession numbers KT582106, KT582107, KT625490, KT692934 and KT714074. A total of 19 SNPs *(single nucleotide polymorphisms)* were identified in exons of the *dhps* gene (Table 4). Of these, 12 SNPs led to silent mutations for all the isolates studied. Of the silent mutations, two have already been described in the literature (codons 664 and 711) and ten are new variants (codons 59, 82, 99, 134, 188, 306, 319, 454, 460, and 547) (Table 4).

**Table 4:** Polymorphisms identified in exons of the *dhps* gene of *Toxoplasma gondii*.

Exon	Nucleotide position in the DNA	Strain/Isolate	Polymorphism	Codon position	Codon	Abreviation/ Amino Acid	Mutation type	Described in the literature
Exon 1a	861	Type I, TgCTBr03, 07, 08, 11 and 16;	T	39	TCT	S/Serine	Non-synonymous	No
		Type II and III.	C		CCT	P/Proline		
	923	Type I, TgCTBr03, 07, 08, 11 and 16;	C	59	CTC	L/Leucine	Synonymous	No
		Type II and III.	G		CTG	L/Leucine		
	940	TgCTBr08, 11 and 16;	G	65	CGT	R/Arginine	Non-synonymous	No
		Type I, TgCTBr03 and 07;	A		CAT	H/Histidine		
		Type II and III.	C		CCT	P/Proline		
	992	Type I, TgCTBr03 and 07;	C	82	CTC	L/Leucine	Synonymous	No
		Type II, III, TgCTBr08, 11 and 16.	G		CTG	L/Leucine		
	1043	Type I, TgCTBr03, 07, 08, 11 and 16;	C	99	ATC	I/Isoleucine	Synonymous	No
		Type II and III.	T		ATT	I/Isoleucine		
	1148	Tipo II, III, TgCTBr03, 07, 08, 11 and 16;	G	134	CTG	L/Leucine	Synonymous	No
		Type I.	C		CTC	L/Leucine		
	1310	Type I, II, III, TgCTBr03, 07 and 16;	A	188	CCA	P/Proline	Synonymous	No
		TgCTBr08 and 11.	C		CCC	P/Proline		
Exon 1b	1664	Type I, TgCTBr03, 07, 08, 11 and 16;	A	306	GTA	V/Valine	Synonymous	No
		Type II and III.	G		GTG	V/Valine		
	1703	Type I, TgCTBr03, 07, 08, 11 and 16;	C	319	ATC	I/Isoleucine	Synonymous	No
		Type II and III.	T			I/Isoleucine		
	1812	Type I, TgCTBr03, 07, 08, 11 and 16;	C	356	CCG	P/Proline	Non-synonymous	No
		Type II and III.	G		GCG	A/Alanine		
	2108	Type I, II, III, TgCTBr03 and 07;	A	454	CCA	P/Proline	Synonymous	No
		TgCTBr08, 11 and 16.	C		CCC	P/Proline		
	2126	Type I, II, III, TgCTBr03 and 07;	C	460	CAC	H/Histidine	Synonymous	No
		TgCTBr08, 11 and 16.	T		CAT	H/Histidine		
Exon 2	3151	Type I, II, III, TgCTBr03, 07, 08 and 11;	C	547	AAC	N/Asparagine	Synonymous	No
		TgCTBr16.	T		AAT	N/Asparagine		
	3184	Type I, TgCTBr03, 07, 08, 11 and 16;	A	558	GAA	E/Glutamic acid	Non-synonymous	E474D [3,5,7]
		Type II and III.	C		GAC	D/Aspartic acid		
Exon 4	4149	Type I, TgCTBr03, 07, 08, 11 and 16;	G	644	AGG	R/Arginine	Non-synonymous	R560K [3,5,7]
		Type II and III.	A		AAG	K/Lysine		
Exon 5	4888	Type I, TgCTBr03, 07, 08, 11 and 16;	T	664	GGT	G/Glycine	Synonymous	580 Sil^a^ [5,7]
		Type II and III.	G		GGG	G/Glycine		
	4938	Type I, TgCTBr03, 07, 08, 11 and 16;	C	681	GCA	A/Alanine	Non-synonymous	A597E [3,5,7]
		Type II and III.	A		GAA	E/Glutamic acid		
	4967	Type I, II, III, TgCTBr03, 07, 11 and 16;	G	691	GCA	A/Alanine	Non-synonymous	No
		TgCTBr08.	C		CCA	P/Proline		
	5029	Type I, TgCTBr03, 07, 08, 11 and 16;	T	711	GAT	D/Aspartic acid	Synonymous	627 Sil [5,7]
		Type II and III.	C		GAC	D/Aspartic acid		

^a^ Sil: Silent

Strains Type I: RH and GTI; Type II: ME49; Type III: VEG.

Seven of the 19 SNPs led to a change in the protein amino acids (non-synonymous mutation). Of these seven SNPs, three have been previously described in the literature (codons 558, 644, and 681), and four were identified for the first time in this study (codons 39, 65, 356, and 691) (Table 4). The G/A/C polymorphism in codon 65 is located in exon 1a and allows the presence of three different amino acids at this protein position, depending on the isolate analyzed. Type II and III clonal strains presented a proline. Type I clonal strains and the TgCTBr03 and TgCTBr07 isolates presented a histidine, whereas the TgCTBr08, TgCTBr11, and TgCTBr16 isolates presented an arginine. The G/C polymorphism in codon 691 is located in exon 5 and leads to a change from alanine into proline. TgCTBr08 isolate presented proline, while the other isolates and clonal strains presented alanine (Table 4). Interestingly, the atypical Brazilian isolates obtained from newborns presented the same amino acid that Type I clonal strains in the polymorphisms observed in codons 39, 356, 558, 664 and 681, while Type II and III clonal strains presented another amino acid in these positions (Table 4).

The previously described mutation N407D (codon 491 with an alteration from asparagine to aspartic acid) [5] was not identified with all the isolates studied presenting the amino acid asparagine (N) at this position. The previously described mutation A587V (codon 671, with a change from alanine to valine) [7, 3] was not found either, with all the Brazilian isolates presenting alanine at this position. It was not possible to obtain the sequence of exon 4 for the TgCTBr03 isolate.

Overall, 40 SNPs were identified in the intronic regions of the isolates studied. The TgCTBr11 and TgCTBr16 isolates presented one SNP identical and exclusive of these isolates in position 5132 of the *dhps* nucleotide sequence. The TgCTBr03 and TgCTBr07 isolates presented one identical and exclusive SNP in position 3669. The TgCTBr08 and TgCTBr11 isolates presented three identical and exclusive SNPs (positions 2395, 3109, and 5753). The TgCTBr16 isolate presented two exclusive SNPs (positions 2999 and 3126) and the TgCTBr11 isolate presented four exclusive SNPs (positions 3729, 5149, 5162, and 5877). In 16 out of 40 SNPs identified in the introns, the isolates from newborns presented a nucleotide identical to the type I clonal strain, and different from the nucleotide of the type II and III clonal strains (positions 727, 728, 745, 2410, 2462, 2920, 2933, 3140, 3333, 4033, 4231, 4925, 5116, 5142, 5865, and 5906). In four SNPs, the newborn isolates presented nucleotide identical to that of the type II and III clonal strains and different from the nucleotide of the type I clonal strain (2908, 3584, 3702, and 4279).

## Discussion

Despite the fact that the Brazilian *T*. *gondii* strains are genetically and phenotypically different from the type I, II, and III clonal strains found in Europe and North America, very few studies have evaluated the response of the Brazilian strains to treatment with extensively prescribed drugs, such as sulfadiazine. There are only two studies conducted with Brazilian strains of *T*. *gondii* that verified the effect of SDZ [14, 18]. In both the authors found that the susceptibility of *T*. *gondii* to SDZ in *in vivo* models varied according to the parasite strain. However, the genetic factors that could be associated with these differences in *T*. *gondii* isolates obtained from humans had not yet been investigated.

In this study, we analyzed the profile of susceptibility to SDZ of five *T*. *gondii* isolates obtained from the peripheral blood of newborns in Minas Gerais as well as the polymorphisms of the *dhps* gene of these isolates. We also compared the susceptibility profiles obtained in this study with those of the other six Brazilian isolates that had been previously characterized [14].

According to the survival curves, the isolates were found to present different profiles of susceptibility to SDZ. The TgCTBr08 and TgCTBr16 isolates were highly susceptible to treatment because the infected mice that were treated with the different dosages presented survival rates greater than 80%. The TgCTBr08 isolate belongs to the genotype ToxoDB #11 (BrII) and the TgCTBr16 isolate to the genotype ToxoDB #08 (BrIII) [10, 12]. The TgCTBr03 and TgCTBr07 isolates presented intermediate susceptibility because the survival rates of the treated mice varied between 60 and 100%, and did not demonstrate a direct correlation with the SDZ dosage. The TgCTBr03 isolate belongs to the genotype ToxoDB #206, whereas the TgCTBr07 isolate belongs to the genotype ToxoDB #67. Treatment with SDZ considerably increased the survival rate of mice infected with isolates TgCTBr03, TgCTBr07, TgCTBr08, and TgCTBr16. These results show that these four isolates are sensitive to treatment at the dosages used.

The TgCTBr11 isolate presented a differentiated phenotypic profile. After infection, the survival rate of treated mice varied between 10% and 40%, suggesting a positive correlation between the SDZ dose and the survival rate. No statistical difference was observed when the survival curves of the groups treated with the different dosages were compared to that of the NTC group. Thus, the TgCTBr11 isolate likely displayed a profile of resistance to SDZ at the dosages used in this study when compared to the other four isolates.

*T*. *gondii* isolates resistant to SDZ were previously described in the literature [5, 7]. This is the first report of *T*. *gondii* obtained from a Brazilian newborn with a profile of resistance to SDZ. Earlier studies used different methodologies to evaluate the phenotype of susceptibility of *T*. *gondii* and to classify it as susceptible or resistant to the treatment. A study from the UK [5] identified a clinical isolate of human toxoplasmosis resistant to SDZ. In a French study [7], three resistant isolates were identified. In this study, we used the methodology proposed by a Brazilian study [14], with modifications, in which tachyzoites were i.p. inoculated in *Swiss* mice and were orally treated with different dosages of SDZ (from 40 to 320 mg/Kg/day) after 48 hours and for 10 days. *In vivo* assays with a murine model had been previously proposed to evaluate the activity of different drugs against tachyzoites of *T*. *gondii* [19].

The isolates used in this study were obtained from the peripheral blood of newborns by mouse bioassay, before starting treatment. The blood was collected after the confirmation of the infection through the detection of anti-*T*. *gondii* IgM antibodies using serological methods. The newborns were 41 to 78 days old on the date of blood collection [10]. The mothers of these infants did not receive any treatment during gestation because the diagnosis was made at the infants’ birth. So, it is possible that the resistance observed for the TgCTBr11 isolate might not have been induced by previous SDZ use. This information is relevant because previous studies have shown that it is possible to induce resistance to SDZ under laboratory conditions by selective pressure [5, 20]. These data support the hypothesis that the TgCTBr11 isolate has a profile of natural resistance to SDZ.

The therapeutic scheme used to treat these infants was a combination of SDZ, pyrimethamine, and folinic acid, according to the dosage per Kg over 12 months [21]. All of the infants showed a good clinical evolution after the treatment, except for the one infant infected with the TgCTBr11 isolate. The clinical data indicate that despite being treated, the newborn from whom the TgCTBr11 isolate had been obtained presented severe congenital toxoplasmosis, ultimately leading to death [10, 21]. At birth, the newborn presented a marked vitreitis and convulsions. The treatment was initiated immediately after screening diagnosis, six days after birth. Three months after the beginning of the treatment, the infant presented active retinochoroidal lesions, vitreous opacification, bilateral retinal detachment, hydrocephalia, microphthalmia hepatosplenomegaly, and convulsions. The infant died at 4.5 months of age due to congenital toxoplasmosis. The severity of toxoplasmosis in this infant may be associated, among other factors, to the low response of *T*. *gondii* to the therapeutic procedure used. The clinical isolate resistant to SDZ from a British patient was also obtained from a severe case of toxoplasmosis, which led to the death of the patient [5].

The number of brain cysts in the surviving mice was also used to verify the efficacy of the treatment. However, the number of cysts did not vary with the SDZ dosage (data not shown). The bioassay with the TgCTBr16 isolate showed that some of the treated animals were infected, despite the fact that brain cysts were not visible by optical microscopy. These results indicate a low parasitism in the brain of the treated animals, also showing that ELISA, compared with the bioassay, has greater sensitivity in the identification of mice infected with the TgCTBr16 isolate.

Although the TgCTBr08, TgCTBr11 and D4 isolates belong to the same genotype (ToxoDB #11), their susceptibility profiles were different. These results corroborate the study conducted in France, which did not find association between susceptibility to the drug and the strain’s genotype when investigating *T*. *gondii* isolates [7]. Studies of Brazilian *T*. *gondii* isolates showed that alleles type I and II at the CS3 locus (located on chromosome VIIa) are strongly linked to parasite virulence in mice, whereas allele type III at the CS3 locus is absent on the virulent isolates [8, 9]. However, isolates with the same susceptibility profile presented different degrees of virulence suggesting that there is no association between susceptibility to SDZ and the allele type at the CS3 locus, or the virulence-phenotype in mice. However, a larger number of atypical isolates need to be evaluated to confirm these hypotheses.

The resistance of *Plasmodium* and *T*. *gondii* to SDZ was previously associated with mutation points of the *dhps* gene [5, 7, 22, 23]. Our study identified 19 SNPs in the exon regions of the *dhps* gene. Five of them had been previously described in *T*. *gondii* isolates from Europe [5, 7] and 14 are new SNPs that are described by the first time in this study. This is the first study showing a large quantity of SNPs in the *dhps* gene of *T*. *gondii*. The larger number of polymorphisms may be a characteristic of the Brazilian atypical isolates because they are genetically different and more diverse compared to those found in North America and Europe [8, 16]. Seven mutation points, leading to a change in the DHPS protein amino acids, were observed in the Brazilian strains isolated from infants with congenital toxoplasmosis. In five of these mutations, the amino acid presented by the Brazilian isolates was identical to the amino acid presented by the Type I clonal strains (GT1 and RH). It is possible that the *dhps* gene of the Brazilian isolates is more similar to the *dhps* gene of the Type I clonal strains than to that of the Type II and III clonal strains.

Two (P691A and H65P) of the seven non-synonymous mutations were observed only in Brazilian *T*. *gondii* isolates, but not observed in the clonal strains. However, although the enzyme DHPS of these isolates may have undergone some alteration in its structure due to this mutation, such an alteration is likely not related to an increase in resistance to SDZ because the mutations under consideration were identified in isolates with a profile of susceptibility to this drug. Mutations N407D and A587V, which were previously described in the literature as being responsible for the phenotype resistant to SDZ in different isolates [5, 7], were not identified in the isolates analyzed in the present study. No non-synonymous mutation exclusive to the TgCTBr11 isolate was identified that could be associated with the low susceptibility of this isolate to SDZ. These results corroborate a recent study conducted in France, which found no association between resistance to SDZ and polymorphisms of the *dhps* gene [3]. Based on the methodology proposed in the literature [5] and reproduced in other studies [3, 7], there is no evidence that there is an association between polymorphisms in the *dhps* gene of *T*. *gondii* and susceptibility to SDZ. A larger number of atypical isolates of *T*. *gondii* must be evaluated to confirm these results.

Other factors may be related to resistance to SDZ, such as the super-expression of proteins critical to the development of the parasite. The ABC transporter superfamily (ATP-binding transporters) is an important family of membrane proteins involved in the resistance to drugs and other biological activities [24]. However, a recent study performed with two induced-resistant *T*. *gondii* strains and three naturally *T*. *gondii* sulfadiazine resistant strains showed that resistance is not related to the overexpression of ABC transporter genes (TgABC.B1 and TgABC.B2) [3].

Another study attempted to identify proteins that are differentially expressed in three sulfadiazine-resistance strains of *T*. *gondii* [25]. The authors identified 31 proteins that are differentially modulated between sulfadiazine resistant and sensitive strains of *T*. *gondii*, according to their genotype. Although none of them allowed a direct identification of the resistance mechanisms to sulfadiazine, the authors suggested that several of these proteins may be associated with the resistance phenotype. Further studies are necessary to verify whether these proteins or other unknown factors could be involved with different phenotypes of susceptibility to SDZ, specifically in Brazilian clinical isolates of *T*. *gondii*.

## Conclusions

This is the first study that evaluates the susceptibility to SDZ of *T*. *gondii* obtained from newborns with congenital toxoplasmosis in Brazil, and verifies whether the identified profile of susceptibility is related to mutations in the *dhps* gene. It is also the first study that evaluates the association between the genotype or virulence-phenotype and the susceptibility profile of the parasite. This study confirms the existence of a Brazilian *T*. *gondii* isolate obtained from human infection, which is resistant to SDZ. We found that the profile of susceptibility to SDZ is probably not associated with the presence of polymorphisms in the *dhps* gene. Further studies, using a large number of Brazilian *T*. *gondii* isolates, must be conducted to confirm these findings.

These results, combined with clinical data regarding a newborns’ response to treatment and a greater knowledge regarding the phenotype, genetics, and population structure of *T*. *gondii* in Brazil, may help establish a more effective therapeutic scheme in the treatment of toxoplasmosis.
